# Role of small private drug shops in malaria and tuberculosis programs in Myanmar: a cross-sectional study

**DOI:** 10.1186/s40545-021-00335-6

**Published:** 2021-11-16

**Authors:** May Me Thet, Myat Noe Thiri Khaing, Su Su Zin, Sandar Oo, Ye Kyaw Aung, Si Thu Thein

**Affiliations:** Strategic Information Division, Population Services International Myanmar, No. 16, West Shwe Gone Dine 4th Street, Bahan Township, 11201 Yangon, Myanmar

**Keywords:** Private drug seller, Community drug shops, Health system engagement, Tuberculosis, Malaria

## Abstract

**Background:**

The role of community drug shops in providing primary care has been recognized as important in Myanmar as in other countries. The contribution by private community drug shops to National Tuberculosis case notifications and National Malaria testing and positive cases is significant. Population Services International Myanmar (PSI/Myanmar) has been successfully training and engaging community drug shops to screen presumptive Tuberculosis to make referrals to public health clinics and perform malaria rapid diagnostic tests (mRDT) to malaria fever cases and provide management accordingly.

**Objectives:**

The study aims to identify barriers to service provision of the trained providers at the drug shops that are currently engaged in PSI/Myanmar Tuberculosis and malaria programs. Exploring their needs enabled us to identify and address barriers, to provide evidence for better linkage with the primary care system.

**Method:**

A mixed method study was conducted with the service providers at the drug shops. A quantitative follow up survey was done with 177 trained Tuberculosis service providers and 65 trained malaria service providers. A total of 32 qualitative in-depth interviews were completed. Seventeen Tuberculosis trained providers and 15 malaria trained providers participated in individual interviews. Content analysis approach was used to generate themes for the data analysis.

**Results:**

From the survey, the majority of drug shops reported that they performed appropriate first steps, particularly referring symptomatic Tuberculosis cases and offering mRDT testing to fever cases. Nevertheless, in-depth interviews with them revealed they did not adhere to the national guidelines for every client. There was a need to emphasize the importance of following the national guidelines for referring patients with prolonged cough and fever cases management. For those who were trained in Tuberculosis case referral, support from program staff was needed to make smooth referrals. Those who were trained in malaria often considered differential diagnosis of fever other than malaria and did not test with malaria rapid diagnostic test due to declining numbers of malaria cases.

**Conclusion:**

The study findings highlighted that the drug shops trained in Tuberculosis referral seemed to have the potential to fully engage into the primary care health system if provided with suitable support and supervision. On the other hand, those trained in malaria case management might be less motivated to engage in the era of declining malaria endemicity.

**Supplementary Information:**

The online version contains supplementary material available at 10.1186/s40545-021-00335-6.

## Introduction

In Myanmar, community drug shops serve as one of the first points of contact for people seeking health care services [1]. These drug shops are often small retail pharmacies located in community and sell prescription and over-the-counter drugs. These drug shops are run by both certified and non-certified personals. In urban and peri-urban areas, they are well regulated by local area health department. However, regulation is not enforced strongly in rural areas and the regular assessments are not conducted.

Over the years, the high burden of Tuberculosis (TB) and malaria have put some stress on Myanmar Health System. TB prevalence rate was 479 per 100,000 population in 2018 [2]. In the same year, 76,518 mRDT confirmed malaria cases were reported [3]. A significant contribution is made by private community drug shops to National Tuberculosis case notifications and National Malaria testing. Population Services International (PSI) Myanmar’s drug seller-initiated program has been one of the channels that has supported National Tuberculosis and National Malaria programs [1, 4].

### Active case finding initiative for Tuberculosis

**Fig. 1:**
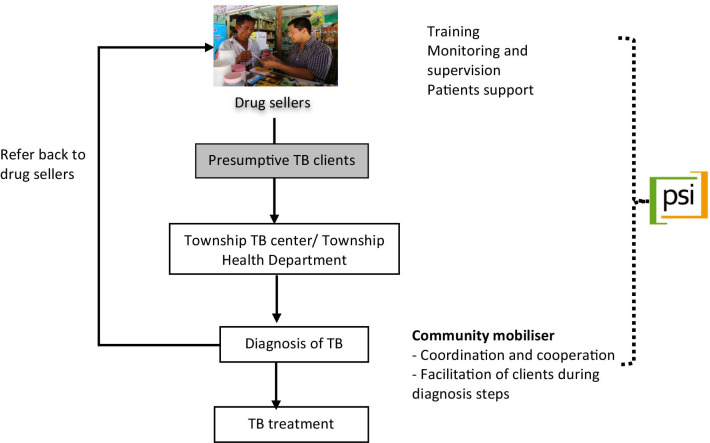
PSI Myanmar TB drug seller model. The arrows indicate the flow of patient from referral phase to treatment and to redirection pathway to the drug shops. The dotted line shows the support that PSI Myanmar is providing to drug shops

PSI Myanmar’s accelerated TB case finding activity (ACF) through informal drug shops started in 2011 together with an active TB case finding channel of interpersonal communicators that focused on actively finding case in communities by providing health education and screening. Since 2011, with international donor support, PSI Myanmar has provided presumptive TB referral by drug shops, where drug shops are trained to refer, record, and report presumptive TB patients. In that model, PSI covers all drug shops in the project townships, especially those located in urban slum areas. With the support of the township medical officers (TMOs), PSI Myanmar identified all registered drug shops present in the project townships, recruited drug shops for the project and provided 1-day training. The training covered screening, case notification and referral according to national guidelines [5]. After the training, post-training knowledge assessment was conducted by standardized question set. PSI Myanmar appointed one field supervisor (community mobilizer) per township to support and monitor drug shops as well as coordinate drug shops and township public health referral centres. In 2018, overall, 3365 presumptive TB cases were notified through this channel and referred to the respective public TB treatment centres for sputum testing [1] (Fig. 1).

When a client comes to a drug shop with symptoms such as cough with or without other symptoms, the drug seller screens the client for presumptive TB by determining their clinical history and checks their eligibility for referral. If the patient’s symptoms meet the criteria for presumptive TB, the drug seller completes two referral forms, one from the National TB Program and another from PSI/Myanmar, and gives the patient the forms and two sputum cups for collection of sputum, along with the instructions. Then, the presumptive TB patient presents to the public TB centre for diagnosis [1].

### AMTR program for malaria

The Artemisinin Monotherapy Replacement Project (AMTR) implemented by PSI/Myanmar is designed to rapidly replace the widespread use of oral AMT with quality-assured ACT (Artemisinin combination therapy). This program has been funded by the international donors since 2012. The project targets ‘private sector’ providers that do not generally have ties with government or non-government not-for-profit organizations such as: drug shops, general retail outlets and itinerant drug vendors [6].

PSI Myanmar has been supporting the expansion of these drug shops’ skillsets by providing 1-day formal training sessions. These sessions cover malaria diagnosis with rapid diagnostic testing (RDT) and treatment in accordance with national guidelines, stock and waste management, counselling, and reporting in collaboration with respective National Malaria Control Program (NMCP) focal points [6]. These RDTs have more than 95% sensitivity and 99% specificity and the results can be read 15–20 min after the testing with whole blood [7]. Providers performed these mRDT tests to fever patients for free. Case-management skills of all drug shop providers are routinely assessed using PSI Myanmar’s app-based Quality Assessment tool, Health Network Quality Improvement System (HNQIS). HNQIS is the quality assessment tool developed by PSI and comprised of four modules designed to assess providers’ quality of care, provide feedback to providers and monitor their performance over time. The system also generates automatic schedules to PSI Myanmar technical support team to visit providers according to the quality scoring [8].

This drug shop channel is also contributing to malaria case testing; through the drug shops network of AMTR projects in 2018, 12,105 cases were tested with RDTs and 289 found positive [4].

Acknowledging that the community drug shops play a major role in tackling the global TB problem, the World Health Organization (WHO) and the International Pharmaceutical Federation (FIP) published a joint statement on stronger engagement of such providers with the national TB Programs [9]. Internationally, studies have been conducted to assess the feasibility, performance and potential of drug shops in providing TB care in community setting. Quality of service and scalability remain challenges for pharmacy-initiated interventions and global attention was needed for improvement [10]. Mixed findings about the performance of drug shop referral service were seen in previous studies. While these drug shops could not provide promising outcomes in some countries [11, 12], some worked well in referring presumptive TB cases, and therefore, sustainability should be considered for the longer term and collaborative actions were suggested [13]. Great opportunities of these providers from TB case finding to treatment completion were reported [14–17]. Existing studies have shown that barriers to drug shops’ performance being drug shops’ reluctance to refer long-standing clients to some DOTS clinics with poor and inadequate service, concern of infection to themselves, their family and other customers when counselling symptomatic patients, fear of client loss, and financial loss and absence of commitment from any PPM collaborating partners. In addition, clients’ attitude such as the hesitance of clients to get medical advice from drug shop providers who did not have proper pharmaceutical training, the patients’ lack of health knowledge of TB and drug shops’ lack of proper pharmaceutical training were found to influence drug seller’s behaviours [12, 16, 18, 19].

Since drug shops are a commonly used source of drugs for fever and malaria, recent policy has included them in malaria elimination strategies; there is extensive literature on the role of these providers in malaria care and treatment and how they could be improved to provide quality care in communities [20, 21]. Past literature shows that drug shops’ behaviour was determined by their knowledge of medicines, consumers’ pressure, their willingness to serve the community, to be seen as respectable, trustworthy citizens, or to be viewed as professionally competent by their fellow drug shops, pricing, profitability, and seasonality [9, 20, 22, 23]. Although there was a lack of firm regulations, drug-shop-based treatment was an important source for public health care and private–public collaboration could improve their capacity and service quality in resource limited settings [24].

Previous findings also suggest that the main reason for drug shops dispensing anti-malarial without prescriptions was the patients’ inability to afford consultation fees, inaccessible and inconvenient government healthcare facilities and the lack of implemented regulations regarding rational dispensing at community pharmacies [25]. Thus, customer demand influences the drug seller adherence to national guidelines in antimalarial treatment. Retail providers’ training and education was found to be associated with knowledge and consequently medicine dispensing behaviours of these providers [22, 26–28].

The objectives of the study were to assess barriers to successful TB and malaria service provision according to national guidelines among providers at the drug shops, to identify their support needs and to explore the potential for linking them with the formal primary care system.

## Methodology

A mixed-method study was conducted with the drug shops currently engaged in the PSI Myanmar TB and malaria programs. First, a cross-sectional quantitative survey was conducted with the trained drug shops. Then, qualitative in-depth interviews were done with a subset of those who had taken part in the quantitative survey. This approach was chosen, because it could provide breadth and depth for the problem that the study aimed to explore. Each method complements each other to better understand why drug shops providers have particular practices. Behavioural studies have used similar approach to explore behaviours and reasons behind them [29–33].

### Study sites

The study sites included 44 townships in 10 states and regions across Myanmar. Specifically, drug shops engaging in TB program were in Ayeyarwady, Bago (West), Magway, Mandalay, Sagaing, Yangon, Mon and those engaged in malaria program were working in Bago (East), Sagaing, Kachin, Kayin, Mon and Tanintharyi.

### Study participants

Inclusion criteria for the study were (i) those who have received training within 1 year before the survey (ii) those who were providing either TB or malaria service at the time of the study. Exclusion criteria were those who received training more than 1 year before the study.

### Sampling and sample size

#### Quantitative survey

**Fig. 2:**
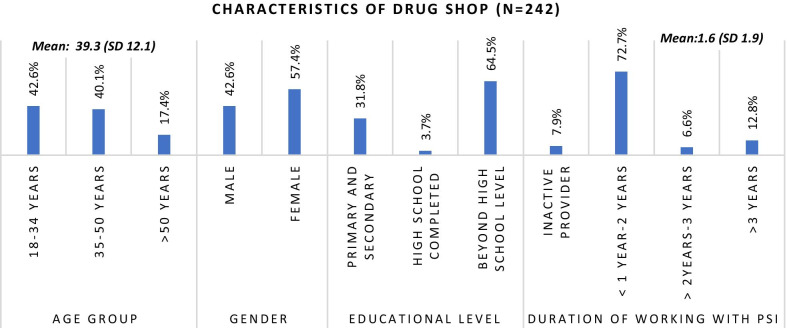
Characteristics of survey participants

**Fig. 3:**
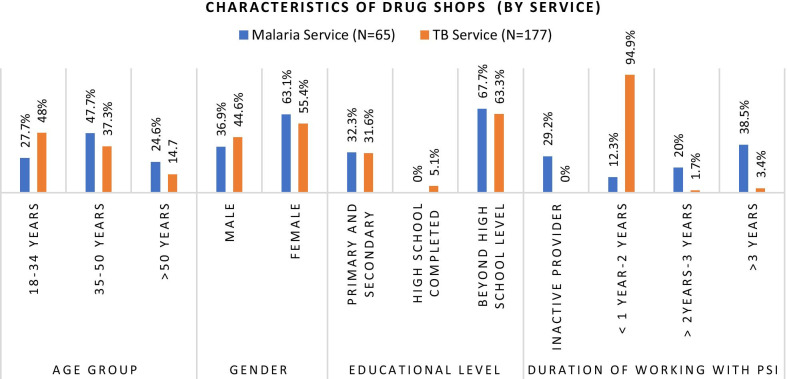
Characteristics of survey participants (by service)

**Fig. 4:**
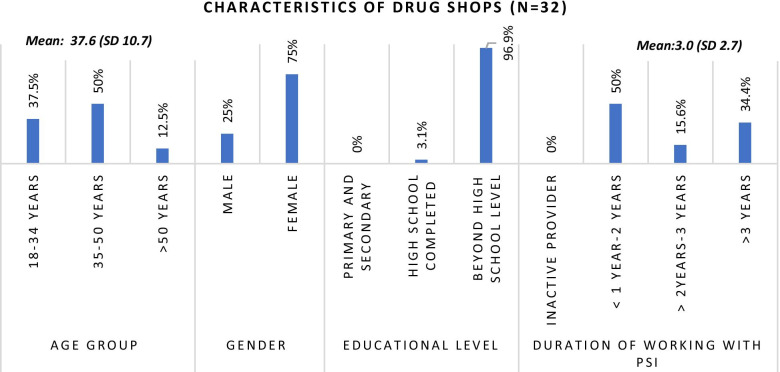
Characteristics of in-depth interviews participants

**Fig. 5:**
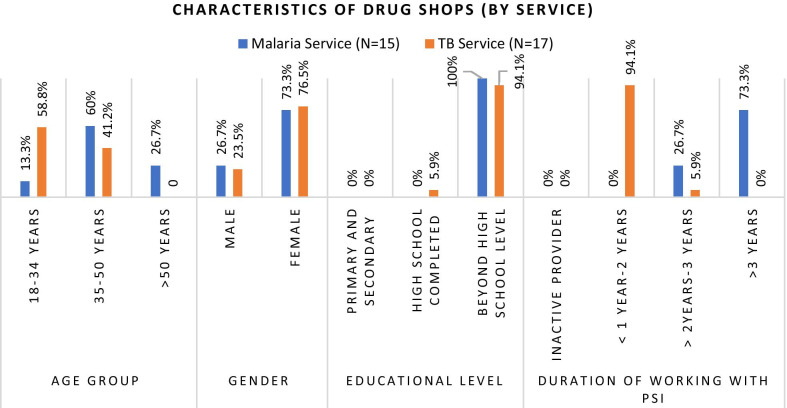
Characteristics of in-depth interview participants (by service)

For the quantitative survey, the list of drug shops engaged in both TB and malaria programs were used as sampling frames. From each list using a systematic random sampling approach, 210 TB drug shops were selected, and 80 malaria drug shops were selected. Among them, 177 drug shops from TB programs in five geographic regions and 65 drug shops from Malaria programs in three geographic regions completed the interviews. Characteristics of participants participated in quantitative survey and qualitative interviews are described in Fig. 2, 3, 4 and 5.

#### Characteristics of respondents

Among the 242 providers who completed the survey, 177 provided TB services and 65 provided malaria services. 103 (42.6%) were male providers with a mean age of 39.3 years. 156 (64.5%) had attained a bachelor’s degree as the highest education and the mean years of experience working in respective programs was 1.6 years with the range up to 10 years.

#### Qualitative in-depth interviews

For qualitative interviews, purposive sampling was used and a subset of quantitative interview participants, those who gave consent, time for individual in-depth interviews and were willing to participate in the in-depth interviews were recruited. A total of 17 in-depth interviews were conducted with TB drug shops from five geographic regions and 15 with malaria drug shops from three geographic regions, respectively.

#### Data collection tools

For the quantitative survey, a structured questionnaire was developed by PSI Myanmar using past literature and experience. For those who were providing referral services of presumptive TB cases, questions around barriers such as attending training provided by PSI, counselling with patients, sending patients for diagnosis tests, referrals to public health departments and relationship with public health department were asked. For those who were providing malaria services, questions around barriers for attending training provided by PSI, testing every fever case with RDTs, treating cases with quality ACTs, ensuring patients to complete treatment, and stock condition were explored. Then, both types of drug shops were asked what and how PSI Myanmar could support them to stay engaged in the network and continue their activities.

For qualitative interviews, standardized interview guides were developed by PSI Myanmar to explore the barriers faced by drug shops to provide TB and malaria services. The interview guides were semi-structured question guides and designed to elucidate in-depth information from individuals. Semi-structured questionnaires containing both closed and open-ended questions were used to determine knowledge, attitudes and management practices regarding TB and malaria and barriers related to these (Additional files 1 and 2).

Both data collection tools were originally developed in English and translated into Myanmar for use in the field. The tools were reviewed by both program and research teams for checking the validity. In addition, both were piloted and pre-tested before use in the field. The results of pilot test and pre-test were used to modify the wording, phrasing, sequence of questions in the data collection tools for better administration in the actual data collection.

Training was provided to the data collection teams on methodology, data collection tools, ethical principles and informed consent procedures. Interviewers who had experience in both quantitative and qualitative research were involved in data collection.

#### National guidelines

The national guidelines for diagnosis and treatment of Tuberculosis and Malaria approved by respective national programs are used in data collection tools development. The Tuberculosis management guidelines stated that patients with one of the symptoms such as fever, cough more than 1 week or more than 2 weeks, cough with blood, weight loss, chest pain, and history of TB contact should be referred for Chest-X ray examination and Sputum microscopy [34]. The malaria management guidelines stated that patients who have uncomplicated malaria symptoms such as fever with chill, headache, diarrhoeas and travel history should be diagnosed based on the patients’ symptoms, a simple history taking and a few basic clinical observations including temperature measurement, and blood examination using mRDT. Patients who are tested positive for malaria parasites should be treated within 24 h after appearance of fever with effective and quality antimalarial drugs. Patients who are tested negative should be treated with anti-pyretics, analgesics and medicines to relieve the symptoms. No antibiotics should be given to those patients [35].

#### Data collection

Data collection was done between November 2019 to January 2020 by the PSI Myanmar research team at shops or residences of the participants. Written informed consent from participants was obtained using standardized informed consent form for both quantitative and qualitative interviews.

#### Quantitative survey

Face-to-face interviews were conducted using C.S Pro software installed on Android tablets and each lasted for about 20 min. Field data were uploaded to the server daily and data quality checks were done for completeness and consistency of the quantitative data as stated in the study protocol.

#### Qualitative in-depth interviews

Face-to-face interviews were performed and audio recordings and field notes were taken during the qualitative in-depth interviews. Each individual interview lasted between 45 min and 1 h. Immediately after interviews, audio recordings were listened and checked for data quality and completeness as guided by the study protocol.

### Data analysis

#### Quantitative survey

The data were cleaned and analyzed by the PSI Myanmar research team. Quantitative data analysis was completed using STATA 14 (© StataCorp, College Station, TX). The data were descriptively analyzed and reported using proportions and, means with standard deviations (SDs). For key indicator value generation, standard guidelines from National Program were used to determine each indicator. Providers’ perceptions, reasons for non-compliance and practices were also calculated and presented descriptively. Chi-square tests were run to compare the difference in proportions and *t* tests were used to compare means [36, 37]. Statistical significance is set as *p* value 0.05 (Fig. 5).

#### Qualitative interviews

Audio recordings were transcribed verbatim into the Myanmar language and only transcripts included in the manuscript were translated into English language by the research team members. Before data analysis, Myanmar transcripts were read thoroughly and checked against audio recordings to ensure there was neither error in transcription nor discrepancies. The data analysis was conducted by four analysts (MMT, MNTK, SSZ and SO), who were trained in qualitative research methods and analysis. ATLAS.ti Software version 7.1.4 was applied for coding and analyzing the data.

Content analytic approach was applied to analyze data which uses a systematic method to analyze the content of the data. We completed the content analysis through the process of coding and identifying themes or patterns [38]. A coding frame was developed based on the question guides used and discussions with the interviewers. We conducted the data analysis in four stages [39]. The stages are coding of data, checking coded data, grouping of similar codes and theme generation. Throughout the analysis process, memos were used to ensure quality of coding and documenting analyses across different team members to strengthen the validity.

#### Ethical approval

The study protocol was approved by PSI Research Ethics Board, WHO Research Ethics Board and Institutional Review Board-1 of Ministry of Health and Sports Myanmar.

## Findings

### Quantitative survey findings

#### Tuberculosis service drug shops

Almost all providers reported that they made presumptive TB cases referrals. A total of 153 (86.4%) study participants stated that they always referred all symptomatic TB clients using the correct criteria. In addition, 161 (90.9%) participants said they educated patients with presumptive TB and appropriately referred them for sputum collection and DOTS.

#### Malaria service drug shops

Regarding malaria testing, 57 (87.7%) participants reported that they chose clients with at least one diagnostic criteria and referred the case according to guidelines. 48 (73.9%) providers followed the government recommended treatment algorithm. When encountering patients who do not meet criteria for antimalarials, 42 (64.6%) participants said they explained the reasons for not giving an anti-malarial and insist on a rapid diagnostic test if indicated. Among all malaria providers, 59 (90.8%) reported that they would recommend rapid diagnostic testing in patients with fever or other malaria symptoms or for patients who had been in forested areas.

Findings related to adherence to the guidelines are presented in Table 1. And the reasons for not compliance to the guidelines are described in Table 2.

**Table 1:** Key adherence indicators

Key Tuberculosis management indicators (*N* = 177)	Tuberculosis service drug shops, *n* (%)
Drug shops that used correct criteria for determining a client as a presumptive TB case^a^	153 (86.4%)
Drug shops that did correct first response for presumptive TB case^b^	161 (90.9%)

Key malaria management indicators (*N* = 65)	Malaria service drug shops, *n* (%)
Drug shops that correctly defined for a client as a suspected malaria case^c^	57 (87.7%)
Drug shops that correctly managed according to the government recommended treatment algorithm for a malaria negative client^d^	48 (73.9%)
Drug shops that practice correctly for client without RDT testing^e^	42 (64.6%)

^a^Numerator: Number of TB drug shops that used correct criteria for determining a client as a presumptive TB case (*n* = 153); Denominator: Number of TB drug shops that responded the question of the criteria for determining a client as a presumptive TB case (*n* = 177). Drug shops that chose at least one criterion as follows: fever, cough more than 1 week/more than 2 weeks, cough with blood, weight loss, chest pain, and history of TB contact

^b^Numerator: Number of TB drug shops that did correct first response for presumptive TB case (*n* = 161); Denominator: Number of TB drug shops that responded question of the first response action for presumptive TB case (*n* = 177). Correct first response for presumptive TB case means explaining about TB, giving pamphlets, referring to TB diagnosis centre/private clinic after contacting with PSI staff

^c^Numerator: Number of malaria drug shops that correctly defined for a client as a suspected malaria case (*n* = 57); Denominator: Number of malaria drug shops that responded the question for defining a client as a suspected malaria case (*n* = 65). Drug shops that chose at least one criterion as followed for determining malaria suspect case fever with chill, headache, diarrhoeas and travel history

^d^Numerator: Number of malaria drug shops that correctly managed according to the government recommended treatment algorithm for a malaria negative client (*n* = 48); Denominator: Number of malaria drug shops that managed a malaria negative client (*n* = 65). Correct management for malaria negative case: the drug shop would never give an ACT for a negative test under any condition

^e^Numerator: Number of malaria drug shops that practiced correctly for client without RDT testing for malaria (*n* = 42); Denominator: Number of malaria drug shops that managed the client without RDT testing for malaria (*n* = 65). Correct management for client without RDT testing for malaria, explaining why they cannot give an anti-malarial and insist on giving an RDT

**Table 2:** Reasons for non-compliance according to national guidelines

Tuberculosis service drug shops (*N* = 177)	No	%
Reasons for not referring suspected TB cases		
Patients refusal	41	23.2
It was unlikely that they had TB	24	13.6
Providers had no time/were busy	7	4.0
Provider had no experience of referral	7	4.0
Short opening hours	2	1.1
Patients preferred to make consultation with another provider	2	1.1
Providers had no interest to refer	2	1.1
Patients thought that they needed to pay money	1	0.6
Difficulties in making phone contact with PSI staff	1	0.6
The provider was on trip	1	0.6
When patients were taking treatment for other disease, e.g., Ischemic Heart Disease	1	0.6
Does not know/refused	10	5.7

Malaria service drug shops (*N* = 65)	No	%
Reasons for not testing with RDT in patient with fever/other malaria symptoms and had been in a forested area		
Did not have a RDT	3	4.6
Needle fear	1	1.5
Reasons for not giving ACT in *Plasmodium falciparum* positive cases		
A chance that patient might come back in case of complications after taking ACT; did not want to deal with any issue that might happen after giving the ACT to patient	2	3.1
Lack of confidence in how to prescribe ACT	4	6.2
Preferred to refer	4	6.2
“Treatment” of malaria was not their duty/job	3	4.6
Too complicated to explain	2	3.1
Does not know/refused	9	13.9

### Qualitative in-depth interviews findings

#### Non-adherence to the national guidelines

It was found that the drug shops did not always adhere to the national guidelines and discussed how they managed clients with cough or fever.

#### Tuberculosis service drug shops

When patients presented with cough, drug shops gave cough suppressants and various antibiotics as common initial treatments. If symptoms were not relieved, these cases were referred to public clinics through their supervisors (Community Mobilizers). A few respondents said that they determined the history of the cough to differentiate between seasonal flu and tuberculosis to make treatment and referral decisions.

*When they came and asked a drug, firstly I asked "How many days has it been? Also, if they have cough, do they produce sputum? Is the cough accompanied with sore throat?" I hardly gave antibiotics to them. The antibiotic was provided only if it (the cough) had not been recovered after 2 or 3 days. If the patient was not getting better after taking antibiotics for more than two weeks, I referred the patient to Community Mobilizer. Antibiotic was given just for 3 days. If not recovered, I turned over the case to Community Mobilizer. ****(Provider 2, Pakkoku, Magway)***

*I asked the duration of cough. If the cough was not serious, it was just for short moment, which occurred due to seasonal changes. It was like having dry cough or cold cough. I have never seen cases with cough symptoms suggestive of TB.* ***(Provider 3, Sagaing, Sagaing)***

Not all clients who presented to drug shops were screened and referred according to National Tuberculosis Program Guidelines. Specific factors facilitating referral were good therapeutic relationship between client and drug seller, willingness to listen to advice for cough treatment and certainty that client would follow through to receive the diagnostic tests and treatment. They stated that they were also more likely to refer poor clients as the treatment available at the government TB clinic was free. One mentioned that he referred trishaw drivers as they had contact with many people because of the nature of their work.

*Yes, I explained. I even refer more who have good relationship with me. ****(Provider 2, Sataung, Sagaing)***

*Usually, there are a lot of trishaw drivers. They need to be tested for sputum if they have cough. They have to interact with a lot of people. ****(Provider 1, South Okkalapa, Yangon)***

There were particular criteria that they used for identifying patients that need referral. These key criteria were chronic cough, evening rise in temperature and loss of weight. There were additional criteria that they considered for referral; loss of appetite, TB contacts in family, chest pain and bloody sputum. There was a varying degree of cough duration as determined by the drug shops and it was between 1 week and 1 month (Table 2).

*Because they have been coughing for more than two weeks. Some people had coughed up a little blood. Others might have weight loss and fatigue, especially in the evening. If these symptoms were reported for three days, I made referral. ****(Provider 1, Myingyan, Mandalay)***

#### Malaria service drug shops

Drug shops' management of clients presenting with fever started with exploring history of symptoms to decide whether it could be malaria or seasonal flu. They suggested mRDT (malaria rapid diagnostic testing) only if the symptoms were consistent with malaria. If they thought that fever was due to seasonal flu, they recommended drugs like fever reducing medication and/or antibiotics without any blood tests.

*Symptoms of malaria were different from those of flu. When the client with fever came to my drug store, firstly I asked the types of fever; fever with chill and rigor/normal fever/fever like flu? And “Do you vomit after meal?” Then, I asked the client based on their situations. In case, I could ask the client about symptoms of malaria from the pamphlet/treatment guideline chart. In my opinion, if the client had cough and feel sick or discomfort, it could be seasonal flu. ****(Provider 3, Mohnyin, Kachin State)***

Since drug shops did not provide mRDT to all fever cases, they used different approaches in deciding who needed malaria testing such as living in malaria endemic areas, having travel history to endemic areas, specific occupation or past history. Clients with occupations related to forest exposure like workers in rubber plantations or logging worksites were suggested to get mRDT testing, because these people had higher risk of malaria. They performed mRDT only when their clients agreed with their suggestion. However, one said that she did not provide mRDT if the clients who did not have any malaria like symptoms even though they were willing to get mRDT.

*Malaria risk areas were classified in my township. If the client came from these malaria risk areas or the client was working at night in these risk areas and the client got fever, I performed mRDT. ****(Provider 1, Mohnyin, Kachin State)***

*If the client came with high grade fever and had symptoms like malaria, and if the client suddenly got fever after going to the forest, I tested with mRDT in such types of clients. ****(Provider 2, Tanintharyi, Tanintharyi Region)***

*I asked the duration of fever. If the fever was for 2–3 days and the client was living in the forest and there was travelling history, I performed mRDT. I did not suggest them to get tested with mRDT if the clients lived in the village got fever. ****(Provider 5, Mohnyin, Kachin State)***

### Supervisor’s support

#### Tuberculosis service drug shops

Many drug shops reported an important support obtained from their supervisors. They made referrals with the help of community mobilizers (CM). Drug shops linked up their clients with CMs and these CMs took care of the referral process and accompanied the clients to referral centres.

*If I was going to transfer, I had to fill up form first. Then I had to call CM for informing that I would like to transfer a patient the next morning. If CM said okay, I gave the sputum cups to the patients and taught him to spit for sputum collection. The forms are also filled out at once. Then I gave them CM’s phone number at the same time saying that CM would call them tomorrow and he would be waiting at hospital. I made it possible to connect to both although I could not accompany with them. ****(Provider 1, Pathein, Ayeyarwaddy)***

*Sputum cups are provided. I wrote on one cup for morning and another for evening. After that, I called CM only if I did not know much clearly about the procedure. Then CM came to me and he checked everything even the form filling****. (Provider 1, Sagaing, Sagaing)***

### Barriers faced by drug shops

#### Time constraint

##### Tuberculosis service drug shops

There were barriers experienced by drug shops to adhere to the national guidelines. The respondents said they had limited time for individual consultation with some clients. This limited their ability to explain the diagnosis and treatment process. Some reported not having time to accompany their clients when they visited government clinics for diagnosis and treatment, because they would like their clients to have smooth referral though it was not included in their duties.

*So, do you not ask anything in addition to this? I asked only if it (patient condition) was too bad. I did not ask when I had many customers or when I had no time. If I had time, I told them to come back if they had not recovered. I would refer them to find the diagnostic centre. I told them like this. ****(Provider 1, Pathein, Ayeyarwaddy)***

*6 pm was peak hour for us. Many customers came to me to buy drugs. So, it was major challenge for me to explain all people. I also studied hard and learned about each disease in order toto explain many of them in consultation approach. As there were so many customers, normally I am not free after 6 pm****. (Provider 2, Thingangyun, Yangon)***

##### Malaria service drug shops

Some drug shops said that they did not give treatment and referred the mRDT positive cases to government health providers like midwives, because they could not follow up patients to make sure they finished the full course.

*The midwife gives proper instruction. She instructs patients more properly than I do. She does not give a full course of treatment if patients are villagers with low health knowledge. She asks them to come to her once a day for treatment. She goes to the patients’ houses if they did not come to her. Does she go to patients’ houses if they do not come to her? Yes, she does. She goes to their houses and convinces them to take treatment. ****(Provider 5, Tanintharyi, Tanintharyi Region)***

### Declining disease endemicity

#### Malaria service drug shops

In recent years, fewer malaria cases were reported in studied areas. The respondents said that they had not seen positive patients for at least 2 months to 2 years. The declining case numbers made less likely to test every fever case and they tried to differentiate between malaria and seasonal flu. If the patient was having sore throat, cold and cough together with fever, they did not perform mRDT. A few claimed that severe fever cases went directly to clinic instead of coming to their drug stores.

*If the client came to cough and sneezing, I did not provide blood testing for malaria because it could not be malaria. ****(Provider 2, Mohnyin, Kachin State)***

*Unlike before, the number of malaria patients declined. The malaria disease becomes rare.*

*I haven’t provided mRDT and detected any positive cases for about a year. ****(Provider 5, Mohnyin, Kachin State)***

Having no antimalarial drugs in stock was sometimes a barrier in providing treatment. Due to declining case load, some drug shops said that they just kept one or two ACT blister packs due to concern for drug expiration if kept in large amounts. Some reported that they had difficulties in providing treatment for patients testing for *Plasmodium vivax,* because they did not have drugs to treat *Plasmodium vivax*. As a result, they had to refer these fever cases to other health providers.

*I do not ask drugs from them as there is no case. Previously, they gave one blister pack of antimalarial drug, but I gave them back because I was worried that the drugs would be expired in my hand. There was no case for me to use at that time. The drugs can be used in our places. ****(Provider 2, Tanintharyi, Tanintharyi Region)***

### Lack of trust in drug shops

#### Tuberculosis service drug shops

Drug shops also mentioned that there was a lack of trust from their clients as they are not medical doctors or formally trained medical personnel, so it was particularly challenging for them to convince clients who needed a referral. Even if the patients were referred by drug seller, their clients might not directly go to the government clinic for screening and diagnosis and instead, they might consult with Specialist doctors or traditional healers.

*Some people accept. Some people are also busy as they are from rural area. Therefore, they are not willing to go there. They seek treatment from the health provider in their village. They think that they will go to the diagnostic centre only if necessary. ****(Provider 1, Sataung, Sagaing)***

*Have you ever told one or two people about the information? Yes, I have told them that they should go to receive TB diagnostic test. But they did not go there. They just said they would seek treatment from other health provider. I think they said like that because they did not trust my words. ****(Provider 2, Kyaukpadaung, Mandalay)***

### Lack of convenience at public facilities

#### Tuberculosis service drug shops

Some patients preferred specialist doctors compared to drug seller referral channel if they could afford the cost of the services. The reasons for the specialist preference was long wait times or a need for multiple visits at public health facilities, which was major barrier for patients living in remote places as they needed to travel far to reach the clinics and the opportunity cost both from a time and cost perspective was high when visiting public facilities.

*Some people do not want to queue in hospital as it is time consuming. Moreover, they have to contact community mobilizer. They do not want to do this kind of things. They rather want to go to the specialist doctor who gives them priority. ****(Provider 2, Pakkoku, Magway)***

*One patient said that community mobilizer accompanied him while having an X-ray at the hospital. She told X-ray technician in front of him to perform X-ray as priority and the technician replied “Yes”. However, when he went there, he was told to come back at 2 pm. He told me that the technician treated him differently as he was not accompanied by PSI staff. When he told me like that, I felt sad. ****(Provider 1, Pakkoku, Magway)***

*The procedure at the hospital took long time. It did not matter if the patient went there by motorcycle. But he went there by tuk-tuk which he shared with other passengers and he had to take the return with others. Otherwise, the cost would be more than ten thousand Kyats (10,000 Myanmar Kyats is equivalent to 7 USD). It costs ten thousand from Pakkoku to there. When he shared tuk-tuk with someone, it costed only four thousand. As he had such experience, I was sad for him. If I went there together with him, he would not encounter like that. ****(Provider 1, Pakkoku, Magway)***

### Inadequate program knowledge

#### Tuberculosis service drug shops

Some drug shops reported that their knowledge of the program and its benefits was unclear. They were not clearly aware of the referral process details, incentives and benefits to both patients and the drug seller themselves. As most drug shops were not involved in the entire TB symptomatic case referral process, their supervisors (CMs) knew all this information and supported the referred cases throughout the process until the diagnosis was confirmed.

*Then, you give them (the patients) the community mobilizer’s phone number. Yes. Ma…. can explain more. I give her phone number to presumptive cases and tell them to contact her and make appointment with her about the place and time. ****(Provider 2, Thingangyyun, Yangon)***

*I only know that chest Xray is done there. I do not know the rest. As there is a sputum cup, sputum test will be performed. If the test is positive, they will go to NTP. I know about the procedure to that extent. I do not know the rest of the procedure. ****(Provider 2, Pakkoku, Magway)***

*How much did you get if you refer one TB suspect? Previously, 1,500 MMK was given for one TB referral. Later, I think that money is not given like before. I did not notice. ****(Provider 2, Thingangyyun, Yangon)***

#### Malaria service drug shops

Drug shops did not know the exact amount of incentive money given for mRDT testing and positive cases found. One respondent even said that he ordered branded antimalarials privately, because he did not know that PSI supported them for free.

*I have received clothes, umbrella, raincoat, bag, and money. The amount of money depends on the number of blood test I perform. I do not remember the exact amount given. I think it is 8,000 MML. How many tests do you have to perform for 8,000 MMK? I forget. I am forgetful as I got older. I think it is for more than 10 tests. You have to ask them. Moreover, some gifts like umbrella, raincoat, clothes, bags, and handkerchiefs were also given. ****(Provider 2, Tanintharyi, Tanintharyi Region)***

### Patient attitudes and beliefs

#### Tuberculosis service drug shops

Another barrier to guideline adherence was patient attitudes and beliefs. Patients sometimes did not perceive themselves as presumptive TB cases even though they were counselled as such by the drug seller. Due to limited health knowledge, patients thought that cough would disappear if they took cough suppressants. Fever, cough, and fatigue were regarded as common symptoms due to seasonal changes and they were not aware themselves and their family as having risk of TB infection. Even though the providers did not directly use the term “TB” in the discussions, and they suggested patient to take sputum test by giving referral forms. Patient did not want to take the test, and some were afraid to take anti TB medicine if they were diagnosed as TB.

*Some people have a belief that the disease is cured if they buy drugs and take them. ****(Provider 2, Pakkoku, Magway)***

*There are people who do not want to accept being told as a TB. These kinds of people refuse to listen to my words. While some people have knowledge about TB, some do not have any health knowledge. Therefore, their disease become severe. ****(Provider 2, Pathein, Ayeyarwaddy)***

*Yep they got scared. Even hearing the sound of the word “TB”. Some people were scared to take medicine. They asked me to sell just one pill with no additional medicine. As they are scared to take medicine, telling them about TB was worsen for them. They got lost after this. They did not come to me anymore. Instead they asked other people (their kids) to buy drugs. ****(Provider 2, Pathein, Ayeyarwaddy)***

Some respondents mentioned that their clients were not willing to get screened for TB. People were scared of being diagnosed as TB cases as there was social stigma around TB patients in community. Even the free services were available to facilitate the timely treatment, they did not want to participate in screening and diagnosis.

*I think they are scared to be diagnosed with TB. Some people are scared that other people will look down on them. Moreover, some are shy. ****(Provider 4, Hlaing, Yangon)***

*Difficulty was that their face looked upset when I explained about it. Sometimes they did not pay attention and even left the shop without buying any medicine. ****(Provider 5, Insein, Yangon)***

Drug shops said that clients sometimes did not always provide correct information of their medical history especially duration of cough. Some patients reported as recent cough history although they had been taking medicine for several days or weeks. Some patients did not like when they were asked details about their cough or TB screening was suggested.

*Mostly they told the cough was recent. Whenever I asked "Have you had a cough for days? How long has it been?", they said they just had now. I noticed that it was not. If a person bought a cough medicine from me 5–10 days ago and he bought drugs again and again, then I noticed for 2 or 3 more times. If I told them that their cough had been a long time and they replied that it was not too long. It (the previous cough) was gone and this was another attack due to eating sweets or something like this. Even though I want to explain to get screened they did not listen to me. ****(Provider 4, Hlaing, Yangon)***

Few respondents revealed that they did not provide mRDT to certain patients such as infants and children who cried and refused to receive test.

#### Malaria service drug shops

Some clients preferred taking antimalarial drugs to receiving mRDT, because they thought that their illness was not severe. Therefore, they would prefer to take drugs without a formal diagnosis. They sometimes asked for specific brand or generic drugs for fever. Some clients demanded antimalarial drugs like Artesunate without mRDT testing.

*One worker from that worksite cannot be urged to receive mRDT. He insisted on buying antimalarial drugs. Therefore, I did not sell him antimalarial drugs and gave him analgesics instead. That person regards himself as malaria if he suffers from muscle aches and pain. Therefore, he asked me to sell antimalarial drug. ****(Provider 1, Mohnyin, Kachin State)***

*I have to sell about 30 tablets of antimalarial drugs (drugs for one month) when some people come to buy them as they have to go to work in the forest for one or two months. ****(Provider 4, Mohnyin, Kachin State)***

They said that some clients who lived in remote areas asked other people to buy drugs especially antimalarial drugs, because they were busy or working and could not come to the shop. This was common among workers engaging in forest related jobs and they could not leave their workplace as they were paid on daily basis.

A few drug shops explained that clients were concerned about side effects of antimalarial drugs. They were afraid of side effects like severe vomiting, nausea, and lethargy, because they had either experienced them or heard of others experiencing these side effects. Therefore, they were reluctant to receive antimalarial treatment despite testing positive.

*Some people have not taken treatment. However, they think that the drugs’ effect is strong. In fact, although the quantity of drugs is large, the effects are not as strong as they think. I tell them to take two drugs in the morning and two in the evening. When they ask me whether the energy weakens, I have to explain them that the drugs do not weaken people. ****(Provider 4, Mohnyin, Kachin State)***

#### Malaria service drug shops

Despite drug shops’ instruction and recommendation on the dosage for treatment, clients usually stop taking antimalarial drugs when they feel better. They perceive that they were cured. A lack of knowledge about malaria disease progression, such as cerebral malaria, was one factor contributing to not completing a full course of drugs. Although the drug shops instructed clients on dosing frequency and duration, their patients reportedly forgot about drugs or left them at home after the first day of treatment.

*When we have a look in a big picture, it is rare to find a person who completes a full course of treatment. They no longer take drugs if their symptoms are relieved. They say that they are cured and well now. It is like that. ****(Provider 2, Mohnyin, Kachin State)***

## Discussion

To our knowledge, this was the first study in Myanmar to investigate the private sector community drug shops’ role in providing symptomatic tuberculosis referrals, and case management of malaria febrile cases. Understanding the drug shops’ performance, barriers and facilitators provided important insights to consider for both Programmers and Policy makers for longer term program achievements and engagement into the primary health system particularly in a setting, where private sector requires strengthening [40].

### Quantitative survey findings

In the study, majority of trained drug shops reported that they could identify presumptive tuberculosis cases and followed the Tuberculosis Referral Guidelines that they explained and made referrals of presumptive cases for diagnosis and treatment. The results were similar to studies conducted in countries with high Tuberculosis burden [15, 16] which applied quantitative methodology. Most malaria trained providers were also able to correctly identify suspected cases. In addition, they reported that they would perform RDT testing for those patients with symptoms of malaria and history of forest travel and practiced as according to national guidelines for case management. The majority of malaria trained drug shops said that they trusted mRDT and use of clinical judgement to guide the case management was not common in their practice. Similar quantitative findings were found in other countries which demonstrated that correct practices were seen in pharmacies that were trained for malaria case management [22, 27, 28]. This finding contradicts that of a qualitative study in Myanmar which reported that medical drug representatives prescribed anti-malarial to patients tested for mRDT negative [41]. Although high rates of symptoms identification and management guidelines adherence are revealed in the quantitative survey, these findings would be complemented by insights from qualitative phase of the study to reflect the complete picture of the drug shop providers’ practices.

### Qualitative interviews

The study shows that the trained drug shops do not always follow the national guidelines. Some used their own judgement rather than adhering to the guidelines, this provides an important insight and highlights the need for supportive supervision, and continuous learning to improve their knowledge especially related to screening and testing for TB and malaria. And that patients presenting with cough alone need diagnostic screening for Tuberculosis and testing of every fever cases with mRDT is important for breaking the malaria transmission chain. Barriers faced by these providers included their time constraint and low awareness on comprehensive program information and benefits. This highlights that there is a need to provide information on treatment pathway, incentives scheme and program benefits to the providers, while they are engaged in the program. In this specific context, their supervisors could be the immediate source of information when they manage a client. The findings suggest that a possible focus in future is having a well-designed package of introduction session together the training to make the drug shops perform better as to the national guidelines.

It was also found that drug shops obtained support from their supervisors who were also PSI staff to make successful referrals. Like in earlier studies, some people considered public tuberculosis clinics as poor quality and provide less-friendly services [18, 42]. Therefore, patients needed support from PSI staff or drug shops to accompany them on clinic visits for smooth process at the public facilities. Due to complex procedures, prolonged waiting time, need of multiple visits at public health facilities were reported in the study. These drug shops relied on PSI program staff and this finding suggested a significant role of program support in community drug shops’ referral channel. Consequently, this will be a factor to consider balancing investment in program support and benefits from the aspects of sustainability of drug seller-initiated referral system. Further research incorporating cost factor might be able to provide more information.

The greatest barrier for TB drug shops performance was patients’ knowledge, attitude and beliefs. Limited health knowledge of patients made the drug shops non-compliance to the national guidelines. Patient’s lack of trust towards the community drug shops was a reason for non-compliance with their instruction. Furthermore, this finding indicated that the providers may dispense antibiotics for treatment of fever and/ cough in addition to non-compliance with the national guidelines. Rationale for the prescription of antibiotics as well as guidance and regulation of doing so are critical for community pharmacies [43, 44]. Thus, their position in health system with regards to this particular issue should be made highly aware in training programs. Other important patient attitudes found in the study were social stigma and fear of tuberculosis. TB is considered as the disease of disadvantaged and poor in Myanmar [45]. Having a belief that cough is only a minor symptom caused patients to avoid testing and diagnosis through drug seller channel referral. Patients did not take cough seriously as a symptom, because it was also a symptom of mild illness, therefore, felt that they did not need to consult a provider.

Similarly, some of their patients demanded antimalarials without mRDT testing. This finding was consistent with other studies in Myanmar as self-medication was a common practice in Myanmar [46] as patients bought medicines without any prescription at drug shops and pharmacies and elsewhere [21, 25]. Patients sent another person to buy medicines for febrile illness, since their daily-paid job did not allow them travel and they preferred self-medication. As the patients missed to receive referral and/or diagnosis, that could result delayed in treatment initiation and contact tracing in Tuberculosis patients and proper management by antimalarials in malaria patients. These patient factors provided knowledge that community knowledge and attitude should be improved. As community based programs were found to be effective in raising awareness of TB health knowledge and correcting perceptions in low resource settings [47, 48], patients could be educated through using approaches like using community engagement activities with leaders, support groups within the community and its members. A study in Myanmar found that use of loud speakers improved care seeking practices of patients for malaria [49]. Creating a social media campaign that focuses on improving knowledge and reducing stigma and misconceptions could be used as part of a community-based approach to early detection of the disease as social media usage in Myanmar is high [50]. In addition, current community based and media-based Tuberculosis and Malaria activities of PSI/Myanmar could use findings of this study to adapt their activities and messages.

Nevertheless, from the in-depth interviews with malaria trained drug shops, one major challenge for them was the declining endemicity of malaria in their geographic areas in recent years. In addition, widespread intensified malaria activities of public sector made resulted in a lower malaria case load at their drug shops. Many of them discussed seeing fewer patients consistent with malaria symptoms and testing febrile patients with mRDT seemed to be uncommon in the trained drug shops’ daily practice. The findings showed that the future programs should consider how to ensure the malaria trained drug shops remain integrated in the health system in the light of declining malaria disease burden in Myanmar.

Disease endemicity seemed to be an important factor to motivate these drug shops to adhere to the National Testing and Management Guidelines in the country. Those trained in tuberculosis referral might successfully link with the public primary care clinics if they are provided with proper support and supervision. On the contrary, those trained in malaria case management might be less motivated to engage into the primary care health system if they are trained solely to provide malaria service. Therefore, programs should consider alternative ways to motivate the drug shops to adhere to the national guidelines, especially for malaria elimination.

## Limitations

This study had a few limitations to note. Precautions should be taken to interpret the self-reported rates of correct behaviors for referrals of TB cases and testing of fever cases by the drug shops. As the data collectors were also from PSI, there could be a desirability bias from the respondents, where they might over-report the referrals than what they would have normally done so. In addition, there might be recall bias on answering the practice of the participants when they took part both in the survey and in-depth interviews. Another limitation to note was that all the participants were affiliated with PSI and there may be selection bias of the participants, and other private drug shops with no affiliation were not included in the study.

## Conclusion

Drug shops in Myanmar faced program support related barriers to adhere to the national guidelines while providing Tuberculosis services in community settings. The barriers identified in this study could be addressed by Programs so that these drug shops would better engage with the existing primary care system. However, declining malaria cases could be the major challenge among others for those trained to test, treat and report malaria cases. This situation should be carefully considered in designing support strategies for malaria service drug shops. Therefore, future research focus on costing aspects of the programs for fairer decision making and sustainability.

## Supplementary Information

**Additional file 1.** Qualitative question guides.

**Additional file 2.** Quantitative Survey Questionnaire.

## Data Availability

The data collected for the current study are described within the manuscript in finding section and available in the article. The data are not publicly available due to the data containing information that could compromise participant privacy. The transcripts and quantitative data set will be available from the corresponding author on reasonable request.

## Abbreviations

ACT: Artemisinin combination therapy
AMTR: Artemisinin Monotherapy Replacement
CM: Community mobilizer
FIP: The International Pharmaceutical Federation
HNQIS: Health Network Quality Improvement System (HNQIS)
mRDT: Malaria rapid diagnostic test
NMCP: National Malaria Control Program
PSI/M: Population Services International Myanmar
RDT: Rapid diagnostic test
TB: Tuberculosis
