# Supplementary material for: Role of small private drug shops in malaria and tuberculosis programs in Myanmar: a cross-sectional study
Source: J Pharm Policy Pract. 2021 Nov 16;14(Suppl 1):89. doi: 10.1186/s40545-021-00335-6 (PMC8594090; doi:10.1186/s40545-021-00335-6)
Supplement: Supplementary file 1 — Additional file 1. Qualitative question guides. [file 40545_2021_335_MOESM1_ESM.pdf]

Qualitative Question Guides

Introduction and building rapport

- Please introduce yourself and explanation of why you will be visiting and what you will be doing today
- Why you have In-depth interviews today
- Explain about the study mentioned in the consent form and ask for consent for participation in the study
- Encourage participants to share experience and opinions
- Assure participant of confidentiality
- Advise them about their decision to participate in interviews will not impact their participation in the program

Questions:

1. History of joining PSI
    a. Can you please tell me about how you joined PSI' network?
    b. How long have you been working with PSI and specifically with the TB/ malaria program?
    c. When and how did you receive training on TB case finding/ malaria testing and treatment? How many times did you join PSI training?
    d. Could you please tell me about your routine activities?
2. Knowledge and attitude about TB symptoms and referral (For TB drug sellers)
    a. Do you know the common symptoms of TB? If yes, what are they?
    b. If patients come with cough, what do you usually do in the first visit? Do you ask patient with cough about its duration?
    c. Do you know the TB referral process? If yes, what are they and how do you make sure to follow this process?
    d. Do you refer all patients with cough? **If not refer**, why? When will you refer those patients? **If refer**, which symptoms make you refer? Where, to whom and how do you refer? What do you need to do for referral? (Probe: Fill out any forms? Others?)
    e. In general, what kind of person do you or don't you refer for TB case notification? Why?
3. Practices on presumptive TB referral
    a. Have you ever encountered presumptive TB patients (patients with symptoms of TB/ patients with cough >2 weeks)?
    b. If yes, how many (approximately) of these patients have you encountered in the last month? Encountered and referred? Encountered but not referred?
    c. If not referred, why?
    d. If referred, can you describe how the patients assessed diagnosis and treatment for TB? Were there any difficulties they confronted?
4. Knowledge and attitude about Malaria symptoms, Testing and Treatment (For Malaria drug sellers)
    a. Do you know the common symptoms of Malaria? If yes, what are they?

    b. If patients come with fever, what do you usually do in the first visit? Do you know the standard guideline for malaria testing and treatment? If yes, how do you make sure to follow this process?
    c. Do you test all patients with fever for RDT? **If not test**, why? When will you test with RDT those patients? **If test**, which symptoms make you do so? Who, when and why you test those cases?
    d. Do you give ACT to all patients with positive for RDT? **If not give**, why? When will you treat those patients with ACT? **If give**, which symptoms make you do so? Who, when and why you give ACTs to those cases?
5. Practices on presumptive Malaria Testing and Treatment
    a. Have you ever encountered patients with fever like malaria?
    b. If yes, how many (approximately) of these patients have you encountered in the last month? Encountered and tested with RDT? Encountered but not tested with RDT? If not tested, why?
    c. How many (approximately) of those with RDT positive are given ACTs in the last month? Positive and given ACTs? Positive but not given ACTs? Negative and given ACTs? Why?
    d. Have you ever had patients who did not take full course of ACTs? If yes, why? How do you think such patients could be encouraged to complete the full course of ACT?

6. Motivating factors
    a. What are the factors that encourage you to stay in this program? (Probe: Community interest? Own interest? Support from program? Support from community? Any financial issue? Any market advantage? Any other motivating factors?)
7. Barriers
    a. What do you think are the barriers that demotivate you and decrease your performance? (Probe: Any financial issue? Any problem with clients/ patients? Any issue with community? Any problem with PSI Myanmar? Any problem with township health authorities/ township health staff? Any other issues? How are these barriers overcome? How do you think the barriers can be overcome?)
8. Support
    a. What additional support do you think you need from PSI to encourage you to stay in the drug seller network and increase your performance?
    b. And/or what support do you need more from others apart from PSI, e.g. township health authorities/ township NTP/ VBDC , community, and patients?
9. Benefit
    a. Which benefit do you get from this particular program? (Probe: Any social benefit? Any economic benefits? Any financial benefits? Any other benefits?)
    b. Does finding TB/ malaria patients among your customers lead to more benefits (social, reputation, more awareness of the disease)?
10. Sustainability issues
    a. Do you think you will continue this practice even without support from Community mobiliser or PSI?

b. If the closely monitored activities of community mobilisers are reduced or no longer exist, what difficulties do you anticipate?
c. Which support do you need to overcome these barriers? Will you continue participating in drug seller network?
d. Is there anything that PSI can do so that you can overcome those problems? Can you please describe them?
