# Supplementary material for: Role of small private drug shops in malaria and tuberculosis programs in Myanmar: a cross-sectional study
Source: J Pharm Policy Pract. 2021 Nov 16;14(Suppl 1):89. doi: 10.1186/s40545-021-00335-6 (PMC8594090; doi:10.1186/s40545-021-00335-6)
Supplement: Supplementary file 2 — Additional file 2. Quantitative Survey Questionnaire. [file 40545_2021_335_MOESM2_ESM.pdf]

Quantitative Survey Questionnaire

General Questions

No.	Question	Options
Q001	How old are you?	|__|__| years
Q002	What is your gender?	1 – Male<br>2 – Female
Q003	What is the highest level of education you have attained?	1 – High School<br>2 – Some college<br>3 – Bachelor's Degree<br>4 – Master's Degree<br>5 – Doctoral/advanced degree<br>96 - Other (monastery education, lower than high school level, and vocational training)
Q004	How long have you worked with PSI's malaria/ TB program?	|__|__| years |__|__| months

Malaria Related Questions

Q101	What symptoms do you use to define a suspected case? Select all that apply.	1 – Fever
		2 – Chills
		3 – Headache
		4 – Vomiting
		5 – Diarrhea
		6 – Travel History
		96 – Other (Specify) ------------------------
		98 – Does not know/refused
Q102	Why do you test suspected cases? Select all that apply.	1 – To follow national guidelines
		2 – Because a patient asks for it
		3 – Financial gain
		4 – Because I don't want to miss a malaria case
		5 – Because I want to rule out malaria to better identify the actual problem
		6 – Because I want to support malaria elimination efforts
		7 – Because PSI supports me to test
		98 – Does not know/refused
Q103	If a test is negative, would anything prompt you to give an ACT? Select all that apply.	1 – No, I would never give an ACT for a negative test
		2 – When they have signs of malaria
		3 – When they ask for malaria treatment
		4 – children
		5 – Pregnant women
		6 – Forest goers
		7 – Other high risk groups
		8 – When I don't trust the test
		9 – When I know the patient

		10 – Because I can make money on the ACT
		11 – Worried that I may miss malaria and the patient will become worse
		12 – If the patient took an antimalarial at home before being tested
		13 – Used to have malaria recently (last month and not feeling better)
		98 – Does not know/refused
Q104	If someone came to see you with a fever/other malaria symptoms and had been in a forested area, is there any reason that you would not give them an RDT?	1 – Yes
		0 – No
		98 – Does not know/refused
Q105	If yes, why would you not test them?	1 – no time
		2 – It's unlikely they have malaria
		3 – They refuse
		4 – I have no incentive to test
		5 – No RDTs in stock
		96 – Other (Specify) ------------------------
		98 – Does not know/refused
Q106	If a test is *Plasmodium falciparum* (Pf) positive, is there any reason you would not give an ACT if it was in stock?	1 - Lack of confidence in how to prescribe
		2 – Too complicated to explain
		3 – Prefer to refer
		4 - "Treatment" of malaria is not my duty/job
		5 – ACT does not make margin so much comparing to spending time for counseling
		6 – A chance of the patient might come back in case of complications after taking ACT; I do not want to deal with any issue that might happen after I give the ACT to patient
		7 – No – I would always give an ACT if the test is positive and I have it in stock
		98 – Does not know/refused
Q107	If an RDT gives a negative result, but the client shows symptoms of malaria, would you provide an antimalarial?	1 – Yes<br>2 – No<br>98 – Does not know/refused
Q108	If no: If a test is negative, what would you give?	1 – Nothing
		2 – An antibiotic
		3 – A fever-reducing medication
		4 - oAMT
		98 – Does not know/refused
Q109	If a client demanded an anti-malarial without first receiving an RDT, what would you do?	1 – Insist on giving an RDT<br>2 – Explain why I cannot give an anti-malarial

		3 – Give/sell the anti-malarial requested<br>4 – Offer another type of non-antimalarial drug<br>98 – Does not know/refused
Q110	What are your main concerns about adherence?	1 – Patients stop taking drugs once they feel better
		2 – Patients like to save drugs for another time
		3 – Patients think they know better
		4 – Patients forget to take the drugs
		98 – Does not know/refused
Q111	How much do you trust RDTs?	1 – Trust completely
		2 – Trust somewhat
		3 – Neutral
		4 – Distrust somewhat
		5 – Distrust completely
		98 – Does not know/refused
Q112	How prevalent is treatment on clinic judgment vs. RDT	1 – Very prevalent
		2 – Somewhat prevalent
		3 – Neutral
		4 – Somewhat not prevalent
		5 – Not at all prevalent
		98 – Does not know/refused
Q113	How prevalent is patient demand for different ACT?	1 – Very prevalent
		2 – Somewhat prevalent
		3 – Neutral
		4 – Somewhat not prevalent
		5 – Not at all prevalent
		98 – Does not know/refused

Tuberculosis Related Questions

Q201	What criteria do you use to define a suspected TB case?<br>Select all that apply.	1 – Fever
		2 – Cough more than 1 week
		3 – Cough more than 2 weeks
		4 – Cough with blood
		5 – Weight loss
		6 – Chest pain
		7 – History of TB contact
Q202	What is your first response when you meet a suspected TB case?<br>Select only one answer.	1 – Give/sell medicine
		2 – Explain about TB
		3 – Give pamphlets
		4 – Refer to TB diagnosis center
		5 – Refer to private clinic
		6 – Refer/contact to PSI staff
		7 –Nothing do special
		96 – Other (Specify) -----------------------------
Q203	If refer, how much do you trust patients would go to the pubic TB diagnostic centers?	1 – Trust completely
		2 – Trust somewhat
		3 – Neutral
		4 – Distrust somewhat
		5 – Distrust completely
		98 – Does not know/refused
Q204	If not refer, why would you not refer them?<br>Select all that apply.	1 – no time
		2 – It's unlikely they have TB
		3 – Patients refuse
		4 – I have no interest to refer
		5 – Cannot contact PSI staff
		6 – Don't know about the referral activity
		96 – Other (Specify) -----------------------------
		98 – Does not know/refused
Q205	If a suspected TB client demanded any medicine without first receiving referral to TB centers, what would you do?	1 – Insist on referral to TB centers
		2 – Explain why I cannot give any medicine
		3 – Give/sell the medicine requested
		96 – Other (Specify) -----------------------------
		98 – Does not know/refused
Q206	What are the barriers you most often confront in participating in TB case referral activities?<br>Select all that apply.	1 – Concern about TB transmission
		2 – Patients refuse
		3 – No Time for explaining about TB
		4 – No staff for explaining about TB
		5 – Don't know about the referral activity
		6 – Difficulties in filling the referral form
		7 – Lack of support from health staff
		8 – Lack of support from PSI/PSI staff
		96 – Other (Specify) -----------------------------

Q207	What are the motivating factors to participate in TB case referral activities?<br>Select all that apply.	1 – PSI program support
		2 – Support from public health staff
		3 – Community spirit
		4 – Religious belief
		5 – Appreciation and recognition from community, health staff and/or PSI
		96 – Other (Specify) -----------------------------
Q208	What are the benefits you received by participating in TB case referral activities?<br>Select all that apply.	1 – Monetary Incentives from the program
		2 – Non-monetary Incentives from the program
		3 – Benefit to your business
		4 – Self-satisfaction
		5 – Appreciation and recognition from community, health staff and/or PSI
		96 – Other (Specify) -----------------------------
Q209	How much do you trust TB diagnosis in the pubic TB diagnostic centers?	1 – Trust completely
		2 – Trust somewhat
		3 – Neutral
		4 – Distrust somewhat
		5 – Distrust completely
		98 – Does not know/refused
Q210	What are your main concerns about adherence to TB treatment?	1 – Patients stop taking drugs once they feel better
		2 – Patients stop taking drugs as they are scared of side effects
		3 – Patients think they know better
		4 – Patients forget to take the drugs
		98 – Does not know/refused
